# The direct digital workflow in fixed implant prosthodontics: a narrative review

**DOI:** 10.1186/s12903-021-01398-2

**Published:** 2021-01-21

**Authors:** George Michelinakis, Dimitrios Apostolakis, Phophi Kamposiora, George Papavasiliou, Mutlu Özcan

**Affiliations:** 1 Private Practice, 5 Plateia Riga Feraiou Sqr, 71201 Heraklion, Crete Greece; 2 Private Practice, Chania, Crete Greece; 3 Department of Prosthodontics, School of Dentistry, National and Kapodistrian University of Athens, Athens, Greece; 4 Division of Dental Biomaterials, Center for Dental and Oral Medicine, Clinic for Reconstructive Dentistry, University of Zürich, Zurich, Switzerland

**Keywords:** Intraoral scanning, 3D printing, Dental implants, Accuracy, Review

## Abstract

**Background:**

The purpose of this narrative review was to examine the applicability of IOS procedures regarding single and multiple fixed implant restorations. Clinical outcomes for monolithic zirconia and lithium disilicate restorations produced through a direct digital workflow were reported.

**Methods:**

A MEDLINE (Pubmed) search of the relevant English-language literature spanning from January 1st 2015 until March 31st 2020 was conducted. In vitro studies comparing digital implant impression accuracy by different IOS devices or in vitro studies examining differences in accuracy between digital and conventional impression procedures were included. Also, RCTs, clinical trials and case series on the success and/or survival of monolithic zirconia and lithium disilicate restorations on implants, manufactured completely digitally were included. In vitro and in vivo studies reporting on restorations produced through an indirect digital workflow, case reports and non-English language articles were excluded. The aim was to investigate the accuracy of IOS for single and multiple fixed implant restorations compared to the conventional impression methods and report on the variables that influence it. Finally, this study aimed to report on the survival and success of fixed implant-retained restorations fabricated using the direct digital workflow.

**Results:**

For the single and short-span implant sites, IOS accuracy was high and the deviations in the position of the virtual implant fell within the acceptable clinical limits. In the complete edentulous arch with multiple implants, no consensus regarding the superiority of the conventional, splinted, custom tray impression procedure compared to the IOS impression was identified. Moreover, complete-arch IOS impressions were more accurate than conventional, non-splinted, open or close tray impressions. Factors related to scanbody design as well as scanner generation, scanning range and interimplant distance were found to influence complete-arch scanning accuracy. Single implant-retained monolithic restorations exhibited high success and survival rates and minor complications for short to medium follow-up periods.

**Conclusions:**

The vast majority of identified studies were in vitro and this limited their clinical significance. Nevertheless, intraoral scanning exhibited high accuracy both for single and multiple implant restorations. Available literature on single-implant monolithic restorations manufactured through a complete digital workflow shows promising results for a follow-up of 3–5 years.

## Background

The origins of intraoral scanning technology (IOS) can be traced back in the early 1970’s when Dr Francoise Duret and coworkers pioneered the first dental intraoral digitizer to obtain an optical impression [1] for an indirect restoration. It would take approximately another 2 decades to introduce digital IOS in mainstream clinical dentistry [2]. Since then, the range of IOS applications has expanded from single tooth or implant-supported restorations [3–5] to fixed dental prostheses [6], occlusal devices [7], removable partial dental prostheses [8, 9] or complete dentures [10–12] and maxillofacial prostheses [13, 14]. Nevertheless, a consensus regarding the implementation of IOS in complete-arch edentulous patients rehabilitated with multiple dental implants has not yet, been established [15]. This approach would necessitate the use of a completely digital implant workflow from the planning stage to final fit. This workflow begins with intraoral direct digitization of the soft tissues and the implants’ position and it continues with the laboratory steps of computer assisted design (CAD) and computer assisted manufacturing (CAM). The final prosthesis is then manufactured in a monolithic design from zirconia, lithium disilicate or hybrid ceramic materials [2]. For restorations in the esthetic zone, minimal porcelain layering of the framework material can also be employed to overcome esthetic limitations related to the physical characteristics of zirconia.

The implementation of the direct digital workflow in fixed implant prosthodontics is not without difficulties. Two main contributing reasons to this are identified in the literature, one being the variations in partial and complete-arch digital scanning accuracy of different IOS devices [16] and also the lack of long-term data on the success and survival of monolithic single, partial and complete-arch fixed prostheses [17]. Joda et al. [18] in a systematic review reported that the number of Randomized Controlled Trials (RCTs) on the subject of complete digital workflow is low and recommendations for clinical routine cannot be made.

Newer IOS hardware and software versions are constantly being introduced by the manufacturers that claim improved scanning accuracy, improved user interface and better patient experience. In addition, new monolithic materials with improved mechanical and physical properties are introduced to the dental market claiming better aesthetics and higher long-term success and survival [2, 19].

The aim of this narrative review was to present an overview on the current evidence regarding the implementation of the direct digital workflow in partial and complete-arch edentulous patients rehabilitated with implant-supported prostheses. Moreover, this review attempted to compare IOS accuracy to conventional implant impression procedures, identify the main clinical factors that influence IOS accuracy and report on the success and survival of the monolithic zirconia and lithium disilicate restorations produced with this particular clinical workflow.

## Methods

### Search strategy

An electronic search of publications from January 1st 2015 to March 31st 2020 was conducted. The cut-off point (2015) was selected because the rate of advancement in scanner hardware and software [20] and dental CAD/CAM material science [19] has accelerated in the past 5 years. The search strategy used a combination of free-text words. A MEDLINE (PubMed) search was performed and the search terms together with the number of records returned are shown in Table 1.

**Table 1:** Free text terms used in the search strategy

Search terms	Number of records returned
*Free-text*	
Intraoral scanner AND scanbodies	4
Intraoral scanner AND implants	95
Intraoral scanner AND accuracy	231
Intraoral scanner AND digital workflow	71
Zirconia AND digital workflow	52
Lithium disilicate AND digital workflow	30

This review included randomized control clinical trials (RCTs), prospective and retrospective clinical trials, case series and in vitro studies focusing on intraoral digital implant impression accuracy. In vitro and in vivo studies comparing different IOS devices in terms of scanning efficiency were included. Studies comparing intraoral digitization to conventional implant impressions in terms of accuracy were also included. Reports on the accuracy of fit as well as on the success and/or survival of monolithic zirconia and lithium disilicate restorations on implants, produced through an IOS impression procedure were also identified and included in this review. In vitro and in vivo studies looking into the fit accuracy of restorations produced through an indirect digital workflow (laboratory scanning) were excluded. Case reports were also excluded. The search included only English-language articles. To further identify any missed articles, the reference lists of the included papers were screened.

The following questions were formulated and addressed in this review:

- What is the IOS accuracy in single implant sites.
- How does IOS accuracy compare to conventional impression accuracy in short-span and completely edentulous implant sites.
- What are the factors influencing IOS’s accuracy.
- What is the survival and success rate of monolithic implant-supported restorations manufactured using the direct digital workflow.

## Results

Initial search identified 483 references. After application of the exclusion criteria, 72 references were eligible to be included in this review. Data from these studies regarding the type of IOS used, type of conventional impression used, impression accuracy in μm, type of study, reference scanner used were extracted and are presented in Tables 2, 3, 4, 5, 6 and 7.

**Table 2:** IOS accuracy compared to conventional techniques

References	Indication	Measurement	Study type	Intraoral/extraoral scanner used	Analogue impression type (stone cast accuracy)	Reference scanner	Conclusions
Lee et al. [29]	Single posterior maxillary implant	3D Surface	In vitro (n = 1)	iTero	PVS (aquasil) mono-closed tray	LAVA scan ST	Milled models from IOS scan exhibited SS more vertical displacement of implant analogue position compared to master model in coronal direction
Koch et al. [30]	Single posterior maxillary implant	3D surface	In vitro (n = 1)	iTero	N/A	LAVA Scan ST (master model)	Variations in the milled models resulting from software and scanner error exhibited statistical significance Software, scanner, and milling error were shown to propagate through the digital workflow to the milled model
Mühlemann et al. [22]	Single posterior implants	3D surface	In vivo (n = 5)	iTero (57 μm) Trios (88 μm) Lava COS (176 μm)	Polyether mono closed metal tray (32 μm)	D103i (imetric 3D SA)	The conventional gypsum implant model had the highest accuracy of implant position compared to 3D printed and milled models from IOS scans
Mangano et al. [23]	Single anterior maxillary implant	3D surface	In vitro (n = 1)	Trios 3 (Tr = 22 μm/Pr = 15 μm) CS3600 (Tr = 15 μm/Pr = 11 μm) Omnicam (Tr = 28 μm/Pr = 30 μm) DWIO (Tr = 27 μm/Pr = 27 μm Emerald (Tr = 43 μm/Pr = 32 μm)	N/A	Freedom DOF	Trios3 and CS3600 were SS more accurate compared to other IOS Accuracy of IOS in complete-arch implants is NOT corelated to IOS resolution

**Table 3:** IOS accuracy compared to conventional techniques

References	Indication	Measurement	Study type	Intraoral/extraoral scanner used	Analogue impression type (stone cast accuracy)	Reference scanner	Conclusions
Lin et al. [64]	Partially dentate mandible with 2 implants and 4 different angulations (0,15,30 and 45 degrees)	Distance and angulation	In vitro (n = 4)	iTero	PVS (Aquasil) open tray, non-splinted	Cagenix	The amount of divergence between implants significantly affected the accuracy of the milled casts created digitally. The digital technique was more accurate when the implants diverged more. At 0 and 15 degrees of divergence, the digital method resulted in highly significantly less accurate definitive casts. At 30 and 45 degrees of divergence, however, the milled casts showed either no difference or marginal differences with casts created conventionally
Mangano et al. [44]	Partially edentulous maxilla with 3 implants Full edentulous maxilla with 6 implants	3D surface	In vitro (n = 2)	Trios 2 (71 μm) CS 3500 (47-63 μm) Zfx Intrascan 117-103 μm) Planscan (233-253 μm)	N/A	Iscan D104I (Imetric3D)	CS3500 most accurate IOS but no SS compared to TRIOS Refractory Index of PEEK is better than Titanium
Flügge et al. [43]	Partially dentate mandible with 2 implants Partially dentate mandible with 5 implants (Kennedy 1)	Distance and angulation	In vitro (n = 2)	Trios iTero True Def	N/A	D250	The precision of IOS decreases with longer distances between scanbodies, especially crossing the midline
Fukazawa et al. [45]	Partially dentate mandible with 2 implants (short and long distance)	Distance	In vitro (n = 2)	Trios 2 (7 and 20 μm) Lava COS (27 μm and 80 μm) True Def (17 μm and 60 μm) Kavo ARCTICA (3 μm and 18 μm)	N/A	CMM UPMC 550-Sarat	Trios comparably accurate to Lab scanner and SS more accurate than the other IOS tested For longer distances, IOS accuracy decreases Deviation of up to 100 μm is acceptable
Basaki et al. [21]	Partially dentate mandible with 4 implants (Kennedy 1)	Distance	In vitro (n = 1)	iTero (116 μm)	PVS monophase with custom trays (56 μm)	D810	PVS impressions were statistically more accurate than IOS Implant angulation did not affect IOS accuracy. Milled 3D casts were less accurate compared to stone casts
Imburgia et al. [46]	Partial maxillary arch with 3 implants	3D surface	In vitro (n = 1)	Trios 3 (Tr = 50 μm/Pr = 24 μm) CS3600 (Tr = 45 μm/Pr = 24 μm) Omnicam (Tr = 58 μm/Pr = 26 μm) TrueDef (Tr = 61 μm/Pr = 19 μm	N/A	ScanRider	CS3600 had SS higher trueness compared to other IOS. No SS differences in precision were found Accuracy in the partial arch is higher for all IOS compared to the Full arch situation
Chew et al. [24]	Partial jaw with 2 implants and 2 different depths	Distance and angulation	In vitro (n = 2)	Trios True Def iTero	Polyether mono (custom tray)	CMM Model Global Silver	Conventional impressions had ss less deviation compared to IOS. Implant depth affected IOS accuracy. Angulation did not affect accuracy
Chia et al. [25]	Partial jaw with 2 implants and 3 different angulations	Distance and angulation	In vitro (n = 3)	Trios (31-45 μm) depending on configuration	Polyether mono (custom tray) (18-33 μm) depending on configuration	CMM Model Global Silver	Distortions were found with conventional and IOS imps. Conventional imps in parallel implants had highest accuracy compared to IOS. Angulation affects IOS accuracy
Marghalani et al. [31]	Partially dentate mandibles with 2 implants	3D surface	In vitro (n = 2)	Omnicam (33-55 μm) True Def (27-39 μm)	Polyether mono on splinted implant copings (open tray) (26-53 μm)	Activity 880 industrial scanner	True Def IOS was more accurate but SS difference were not always observed Low deviations < 56 μm
Kim et al. [47]	Partially dentate mandible with 6 implant cylinders	Distance	In vitro (n = 1)	Trios 3 Omnicam CS3600 I500 iTero Element	N/A	StereoSCANneo	All IOSs exhibit deviations as scanning distance increases from the start position Trios3 and Medit outperformed other IOSs for partially edentulous accuracy
Mangano et al. [23]	Partial edentulous maxilla with 2 implants	3D Surface	In vitro (n = 1)	Trios 3 (Tr = 28 μm/Pr = 21 μm) CS3600 (Tr = 23 μm/Pr = 17 μm) Omnicam (Tr = 38 μm/Pr = 43 μm) DWIO (Tr = 49 μm/Pr = 34 μm Emerald (Tr = 49 μm/Pr = 29 μm)	N/A	Freedom DOF	Trios3 and CS3600 were SS more accurate compared to other IOS Accuracy of IOS in implants complete arch is NOT corelated to IOS resolution
Motel et al. [65]	Titanium partial model with 3 implants and 3 different scanbody designs and 2 different scan strategies	Distance and 3D surface	In vitro (n = 1)	Trios 3	N/A	ATOS So4 II	All in One scan strategy produced more accurate results (71 μm) Emergence profile scan produced lower accuracy (125 μm) In All in One scan strategy, cylindrical scanbodies with flatter surfaces produced more accurate results
Alsharbaty et al. [32]	Partially dentate mandibles and maxillae with 2 posterior adjacent implants	Distance	In vivo (n = 28)	Trios 3	PVS (Panasil) dual mix, plastic tray/splinted (used as reference) PVS (Panasil) dual mix, plastic tray/non splinted in open and closed tray methods (used for comparison)	CMM (Dea Global)	Conventional open tray pick-up impression was ss more accurate compared to IOS and conventional closed tray pick-up impression
Jiang et al. [92]	Partial dentate maxilla and mandible with implants and 2–4 teeth span	3D surface	In vivo (n = 31)	Trios (27 μm)	Material not provided/splinted, open tray	D800	The 3D discrepancy between digital and traditional impression is within clinical acceptable range

Partial edentulous implant sites

*N/A *not applicable, *Tr *trueness, *Pr *precision

**Table 4:** IOS accuracy compared to conventional techniques

References	Indication	Measurement	Study type	Intraoral/extraoral scanner used	Analogue impression type (stone cast accuracy)	Reference scanner	Conclusions
Gimenez-Gonzalez et al. [59]	Full arch edentulous maxilla with 6 implants	Distance and angulation	In vitro (n = 1)	Lava COS	N/A	CMM Mitutoyo Crista Apex	Operator experience ss influenced accuracy. Angulation and depth of placement did no ss influence accuracy
Gimenez et al. [48]	Full arch edentulous maxilla with 6 implants	Distance and angulation	In vitro (n = 1)	3D Progress ZFX Intrascan	N/A	CMM Mitutoyo Crista Apex	Experience of the operator, implant angulation, and implant depth were not associated with significant differences in accuracy with either scanner ZFX presented higher FA accuracy
Papaspyridakos et al. [33]	Full arch edentulous mandible with 5 implants	3D Surface	In vitro (n = 1)	Trios 2	Polyether mono Implant level splinted/unsplinted Polyether mono Abutment level splinted/unsplinted	Iscan iD103 Imetric	IOS resulted in accuracy similar to splinted conventional implant impressions. Both were SS more accurate to non-splinted conventional imps. Implant angulations up to 10–15 degrees did not affect IOS accuracy
Vandeweghe et al. [49]	Full arch edentulous mandible with 6 implants	3D Surface	In vitro (n = 1)	Trios 2 (28 μm) Lava COS (112 μm) True Def (35 μm) Omnicam (61 μm)	N/A	104i Imetric	Newer generation IOS performed very well regarding full arch accuracy
Imburgia et al. [46]	Full arch edentulous maxilla with 6 implants	3D surface	In vitro (n = 1)	Trios 3 (Tr = 67 μm/Pr = 31 μm) CS3600 (Tr = 60 μm/Pr = 65 μm) Omnicam (Tr = 66 μm/Pr = 57 μm) TrueDef (Tr = 106 μm/Pr = 75 μm	N/A	ScanRider	CS3600 had SS higher accuracy compared to other IOS. Accuracy in the partial arch is higher for all IOS compared to the Full arch situation
Amin et al. [34]	Edentulous mandible with 5 implants	3D Surface	In vitro (n = 1)	Omnicam (46 μm) True Def (19 μm)	Polyether mono splinted (custom open tray) (168 μm)	Activity 880 (Smart Optics)	Digital IOS FA impressions were ss more accurate compared to conventional FA impressions True Def IOS was ss more accurate than Omnicam IOS
Gimenez et al. [62]	Edentulous maxilla with 6 implants	Distance and angulation	In vitro (n = 1)	True Def (70 μm)	N/A	CMM Mitutoyo Crista Apex	The size of visible scanbody affects accuracy. Angulation of scanbodies does not influence accuracy. Scan distance (full arch) affects accuracy
Ciocca et al. [60]	Edentulous titanium mandible with 6 implants	Distance	In vitro (n = 1)	True Def (41-82 μm)	N/A	OCMM SmartScope Flash CNC 300	Operator experience did not influence mean IOS FA accuracy Deviations increased with increase in the length of scan
Alikhasi et al. [39]	2 Fully edentulous maxillae with 4 implants each (trilobed and external hexagon connection)	Distance and angulation	In vitro (n = 2)	Trios 3	PVS dual mix with custom trays (open and closed tray)	CMM Mistral and CMM Atos Core 80	IOS was ss more accurate than PVS open and closed tray. PVS open is ss more accurate than PVS closed. Type of implant connection does not influence IOS accuracy. Implant angulation does not influence IOS accuracy
Mutwalli et al. [50]	Edentulous maxillary cast with 5 implants	Distance	In vitro (n = 1)	Trios 3 mono (63 μm) Trios 3 (114 μm) iTero (41 μm) Atos Core (19 μm)	N/A	Carl Zeiss CMM	Low precision of all IOS for full arch scanning iTero was statistically the most accurate TRIOS official strategy was not used
Gintaute et al. [63]	Edentulous mandibular models with 4 and 6 implants with different angulations	Distance	In vitro (n = 4)	TrueDef	PVS dual mix PE single step both with custom open tray	CMM Createch Medical	The accuracy of the IOS and conventional impression-making approaches for straight and tilted dental implants was comparable, and might be clinically considered for full-arch, multiple-implant restorations
Tan et al. [37]	Maxillary full arch models with 6 and 8 implants	Distance	In vitro (n = 2)	Trios True Def Ceramill Map400 InEos X5 D900	Polyether mono splinted (open tray)	CMM (Renishaw)	True Def was ss less accurate Conventional imps had better accuracy compared to IOS Decreasing implant distance may help reduce IOS distortion
Kim et al. [36]	Full arch edentulous maxilla with 6 implants	Distance	In vitro (n = 1)	Trios 3	PVS Aquasil mono, custom tray-splinted	Contura CMM	Conventional open-splinted tray impression produced more accurate impressions compared to IOS
Mangano et al. [23]	Fully edentulous maxilla with 6 implants	3D Surface	In vitro (n = 1)	Trios 3 (Tr = 46 μm/Pr = 35 μm) CS3600 (Tr = 44 μm/Pr = 35 μm) Omnicam (Tr = 70 μm/Pr = 89 μm) DWIO (Tr = 92 μm/Pr = 111 μm Emerald (Tr = 66 μm/Pr = 61 μm)	N/A	Freedom DOF	Trios3 and CS3600 were SS more accurate in Full arch compared to other IOS Accuracy of IOS in implants FA is NOT corelated to IOS resolution
Mizumoto et al. [75]	Full edentulous polyurethane maxillary cast with 4 implants	Distance and angulation	In vitro (n = 1)	Trios	N/A	COMET L3D	Accuracy of 4- implants FA is not affected by inclusion of the palate in the scan or not
Rech-Ortega et al. [40]	Model with 6 implants	Distance	In vitro (n = 1)	True Definition (21-118 μm) depending on the interimplant distance	Polyether (open tray) non-splinted 20-68 μm depending on the interimplant distance	CMM Heningshaw	For adjacent implants (up to 4) both techniques are satisfactory The longer the distance between implants, the lower the accuracy of both techniques
Di Fiore et al. [51]	Full edentulous mandibular PMMA cast with 6 scanbodies	Distance and 3D Surface	In vitro (n = 1)	Trios 3 (32 μm) True Def (31 μm) Omnicam (71 μm) 3DProgress (344 μm) CS3500 (107 μm) CS3600 (61 μm) Emerald (101 μm) DWIO (148 μm)	N/A	SmartScope CMM	Some IOS performed better than others in full arch scans The size of the output file is independent of the accuracy of the IOS
Arcuri et al. [61]	Fully edentulous maxilla with 6 implants	Distance and angulation	In vitro (n = 1)	Trios 3	N/A	ATOS Compact Scan 5	Implant scanbody material significantly influenced IOS FA digital impression with peek showing the best results on both linear and angular measurements, followed by titanium, with peek-titanium showing the worst results Implant angulation significantly affected the linear deviations while implant position the angular deviation. No significant operator effect on the IOS accuracy was detected
Bilmenoglou et al. [53]	Edentulous mandible with 6 implants	3D Surface	In vitro (n = 1)	Trios color pod (31 μm) Trios color cart (40 μm) Trios mono cart (43 μm) 3Dprogress(102 μm) Omnicam (32 μm) Bluecam (45 μm) Apollo DI (37 μm) E4D (82 μm) Planscan (345 μm) Lythos (113 μm)	N/A	ATOS CORE 80	TRIOS devices, Omnicam, Apollo DI, and Bluecam are suitable for implant-supported complete-arch fixed dental prostheses
Sami et al. [52]	Edentulous mandibular model with 6 implants	3D surface	In vitro (n = 1)	Trios TrueDef Omnicam Emerald	N/A	Edge ScanArm (Faro)	No statistical or clinical differences were found among the scanners tested. The 3D map was the best method for observing the data
Miyoshi et al. [35]	Maxillary edentulous model with 6 implants	Distance	In vitro (n = 1)	Trios 2 (Pr = 29 μm) TrueDef (Pr = 16 μm) Omnicam (Pr = 19 μm) CS3600 (Pr = 21 μm)	PVS dual mix (Imprint 4) with custom open tray-splinted-abutment (Pr = 21 μm)	D810 (Pr = 3,9 μm)	Range of scanning influenced impression accuracy. Digital impressions for implants should be limited to 3-unit structures on 2 impl
Mizumoto et al. [66]	Edentulous maxilla with 4 implants scanned with 5 different sets of scan bodies and 4 different strategies	Distance	In vitro (n = 1)	Trios	N/A	COMET L3D	Scanbody design influences accuracy (the smoother the better). Also, soft tissue surface modifications (pressure paste) did not produce more accurate scans
Huang et al. [38]	Edentulous mandibular cast with 4 implants and 3 different scanbody designs	3D Surface	In vitro (n = 1)	Trios 3 (Tr = 28-38 μm/(Pr = 27-48 μm) depending on the scanbody used.)	PVS putty and light (Silagum) splinted (open tray) (Tr = 25 μm/Pr = 19 μm)	D2000	Conventional splinted open tray impressions were ss more accurate than IOS digital impressions. Experimental design with interconnected scanbodies SS improved accuracy
Chochlidakis et al. [58]	Full arch maxillary edentulous patients with multiple implants (4–6)	3D Surface	In vivo (n = 16)	True Def (RMS 162 μm) 4 implants (139 μm) 5 implants (146 μm) 6 implants (185 μm)	Heavy and light PVS (Imprint)-open tray technique	7series (Dental Wings)	Mean IOS deviation was 162 μm which is marginally acceptable for clinical accuracy Increasing the implant number tended to increase the global deviation in the IOS impressions but with no SS

Complete edentulous arches with multiple implants

*N/A *not applicable, *Tr *trueness, *Pr *precision

**Table 5:** Studies on accuracy of 3D printed models with multiple implants

References	3D printers tested	Indication	Measurement	Study type	Intraoral/extraoral scanner used	Analogue impression (type)	Reference cast	Reference scanner	Conclusions
Revilla-Leon et al. [42]	Projet 3510 (POLYJET) Prodways Promaker D35 (DLP) Objet Eden (POLYJET) Infinident (SLA)	Maxillary edentulous arch with 7 implants	Distance	In vitro (n = 1)	DS20 (Renishaw)	Polyether, splinted with custom tray	Type IV gypsum (Fujirock) with 7 ELOS analogues	CMM	For the 3d printed models, more distortion was observed in the X axis DLP and POLYJET showed accuracy comparable to stone cast
Papaspyridakos et al. [41]	Form2 Formlabs (SLA)	Mandibular edentulous cast with 4 implants	3D surface	In vitro (n = 1)	Trios 3	N/A	Master stone cast	Activity 880 Smart Optics	the printed casts had a mean SD RMS error of 59 μm The implant 3D deviations of the printed casts from complete-arch digital scans had statistically significant differences compared with those of the master cast but may still be within the acceptable range for clinical application

*N/A *not applicable, *Tr *trueness, *Pr *precision

**Table 6:** Studies on single-implant retained monolithic restorations (complete digital workflow). (N/A = not applicable, Tr = trueness, Pr = precision)

References	No of patients/mean age/follow-up	Indication	Location	Abutment type	Intraoral scanner used	Success (%)/survival (%)	Complications	Conclusions
Joda and Brägger, [86]	20/55,4y/N/A	40 single implant screw retained crowns Test: 20 Zirconia (digital impression) Control: 20 metal-ceramic crowns (conventional impression)	Premolar and Molar-Maxilla and mandible	Test: customised titanium abutments Control: prefabricated abutments	iTero	100/100 for both groups at delivery	No corrections needed at delivery for either group	Mean total production time, mean clinical and mean laboratory time were SS shorter for the test group compared to the control
Joda and Brägger, [87]	20/55,4y/N/A	20 single implant screw-retained crowns Test:10 LS2 crowns (digital impression) Control:10 Zirconia-porcelain crowns (digital impression + model milling)	Premolar and Molar-Maxilla and mandible	Prefabricated Ti-base abutment	iTero	100/100 for both groups at delivery	Test: no corrections needed at delivery Control: 40% interproximal corrections, 30% occlusal corrections	Mean total production time (clinic and lab) was SS shorter in the test compared to the control group Especially the laboratory time efficiency was SS shorter for the complete digital workflow
Joda et al. [84]	20/55y/3y	20 single implant Zirconia-porcelain cement-retained crowns (digital impression + model milling)	Premolar and Molar-Maxilla and mandible	Customised Ti abutments	iTero	100/100	None observed	The patients’ level of satisfaction correlated well with FIPS
Joda et al. [5]	44/58,1y/2y	50 single implant LS2 screw-retained crowns	Premolar and Molar-Maxilla and mandible	Prefabricated Ti-base abutment	iTero	100/100	None observed	CAD/CAM-produced monolithic implant crowns out of LS2 in a complete digital workflow seem to be a feasible treatment concept for the rehabilitation of single-tooth gaps in posterior sites under mid-term observation
Joda et al. [93]	20/55,4y/3y	20 single implant screw-retained crowns Test:10 LS2 crowns (digital impression) Control:10 Zirconia-porcelain crowns (digital impression + model milling)	Premolar and Molar-Maxilla and mandible	Prefabricated Ti-base abutment	iTero	100/100 for both groups	None observed	Subjective patient's perception of posterior implant crowns processed in complete digital and combined analog–digital workflows revealed comparable high levels of satisfaction on the overall treatment outcome including function, esthetics, and cleanability after 3 years
Mangano and Veronesi, [79]	50/52,6y/1y	50 single implant crowns, cement-retained Test:25 zirconia crowns (digital impression) Control:25 metal-ceramic crowns (conventional impression)	Premolar and Molar-Maxilla and mandible	25 Customised Zirconia abutments on Ti bases 25 Customised Titanium abutments	CS3600	92/100 for both groups	Test: 4% biologic 4% prosthetic 0,39 mm bone loss (mean) Control: 8% biologic 0% prosthetic 0,55 mm bone loss (mean)	Identical survival and complication rates between groups No SS differences in marginal bone loss Patients preferred the digital procedures more Digital procedures were more time and cost effective
Joda et al. [83]	20/55y/5y	20 single implant Zirconia-porcelain, screw-retained crowns	Premolar and Molar-Maxilla and mandible	Customised Ti abutments	iTero	95/95	1 implant loss Mean bone loss: 0,23 mm mesially 0,17 mm distally	CAD/CAM-processed implant crowns demonstrated promising radiographic and clinical outcomes after 5 years in function
Mangano et al. [80]	25/51,1y/1y	40 single implant zirconia screw-retained crowns	Premolar and Molar-Maxilla and mandible	25 Customised Zirconia abutments on Ti bases	CS3600	92,4%/97,5%	7,6% prosthetic	Minor complications such as infra-occlusion, interproximal issues, aesthetics, de-cementation of crowns were reported
Delize et al. [78]	31/47,5y/N/A	Single implant screw-retained crowns 31 Zr-Porcelain crowns (conventional impression) 31 Zr crowns (digital impression)	Premolar and molar-Maxilla only	Prefabricated Ti bases	Trios 2	96,8 for Zr digital 100 for Zr-porcelain	1/31 Zr crown could not be seated on the Ti-base abutment at try in No follow-up	Both crowns displayed acceptable and comparable clinical precision (contact points and occlusion) From an esthetic point of view, both the patients and the dentists preferred the conventional over the monolithic Zi crowns
De Angelis et al. [81]	38/65,6y/3y	19 LS2 cad- cam screw-retained crowns (digital impressions) 19 Zirconia screw-retained crowns (digital impressions)	Premolar and molar	Prefabricated Ti-bases	Bluecam	LS2 group: 89/100 Zirconia group: 95/100	LS2 group: 5% prosthetic Zirconia group: 5% prosthetic	Monolithic CAD-CAM lithium disilicate and zirconia screw-retained single crowns fabricated with a fully digital workflow were found to be reliable and suitable clinical options for restoring a posterior missing tooth on a dental implant
Lerner et al. [82]	90/53,3y/3y	106 single implant screw retained monolithic zirconia crowns (digital impression)	Premolar and Molar-Maxilla and mandible	Hybrid zirconia abutments with titanium bonding base	CS3600	91,3/99	1,9% Biologic 5,7% Prosthetic	The quality of the fabrication of the individual hybrid abutments revealed a mean deviation of 44 μm (± 6.3) between the original CAD design of the zirconia abutment, and the mesh of the zirconia abutment captured intraorally at the end of the provisionalisation. At the delivery of the MZCs, the marginal adaptation, quality of interproximal and occlusal contacts, and aesthetic integration were excellent

**Table 7:** Studies on multiple-implant retained monolithic restorations (complete digital workflow)

Reference	Indication	Measurement	Study type	Intraoral/extraoral scanner used	Analogue impression (type)	Reference scanner	Conclusions
Rutkunas et al. [85]	48 two-implant retained zirconia FPDs (2,3 and 4 units)	Distance and angulation Screw resistance Clinical fit	In vivo (n = 24 patients)	Trios 3	PVS (Express) splinted-open tray	D800	Angulation of more than 10° between the implants could negatively affect the passive fit of the digitally fabricated restorations intraorally Inter-implant distance does not seem to affect the passive fit of restorations, independent on if they are made digitally or conventionally

### IOS accuracy in single implant sites

Several in vitro studies were identified examining the scanning accuracy of IOS in single-implant edentulous sites (Table 2). IOS scan accuracy has been studied in conjunction with the digital manufacturing of the master model through rapid prototyping techniques. This approach necessitates the milling or 3D-printing of the master model from the IOS scan in order for the restoration to be completed in a semi-digital approach usually employing a porcelain layering step. Alternatively, a complete digital workflow utilizing a monolithic restoration and without necessitating the fabrication of a physical model can be used. Evidence suggests, however, that neither of these approaches is without discrepancies and that the final implant position in the virtual or the physical master model is statistically significantly different compared to the analogue position in the cast model, produced from a conventional impression [21–23]. Mangano et al. [23] in a comparative study reported discrepancies in the virtual position of a single implant ranging from 15 ± 0.8 to 43 ± 11 μm depending on the scanner tested. Deviations of 7–37 μm in the final vertical position of the single virtual implant were also reported in another in vitro study by Chew et al. [24] and correlated to the implant platform placement depth and the scanner used. In another in vitro study by Chia et al. [25] a 15 N/cm torque, applied during tightening of the polyetheretherketone (PEEK) implant scanbody, was shown to alter the position of the implant as much as 11 (± 4.9) μm in an apical direction due to compression of the scanbody material. The surface matching discrepancies between the scanbody and the implant platform, have also been shown to amount to 9–11 μm [26, 27], further contributing to digital impression total inaccuracies.

Additional implant positional discrepancy can be expected when a physical master model is digitally produced. Revilla-Leon et al. [28] in an in vitro study reported that the design of scanbodies significantly affected the positional accuracy of the implant analogues inside the 3D-printed cast. Mühlemann et al. [22] in an in vivo study reported that the conventional impression and gypsum production procedure exhibited higher positional accuracy (32 ± 11 μm) of the implant analogue compared to IOS and digital model production (57 ± 32–176 ± 120 μm), regardless of the scanner and the rapid prototyping process used for fabrication of the plastic model. The fact that this was an in vivo study and that measurements were made on printed/milled models may have contributed to the un-favorable IOS accuracy results along with operator calibration. Furthermore, Lee et al. [29] attributed the implant positional discrepancy to the inaccuracy of friction-fit placement of the digital implant analogue inside the plastic model. All of the aforementioned factors can lead to the final implant crown being over- or infra-occluded therefore requiring major chairside adjustments [30] or even additional laboratory procedures.

### IOS accuracy compared to conventional impression accuracy in short-span implant edentulous sites

Regarding implant-rehabilitated short-span edentulous sites, several studies have compared the IOS accuracy of various scanner devices to the conventional impression accuracy (Table 3). Digital implant impression for short span prostheses, supported by up to 2–4 implants located within the same quadrant, has been mainly compared for in vitro accuracy to an elastomeric impression technique, utilizing either addition-cure silicone materials in a single or dual mix technique, or a polyether mono-phase technique using custom trays [21, 24, 25, 31]. Implant impression posts, in the conventional impression approach, were usually not splinted. Statistical superiority of the conventional method was reported in the majority of studies but the accuracy deviation of the IOS devices ranged from 27 to 66 μm depending on the scanner, whereas for the conventional method the deviation ranged from 26 to 49 μm [24, 25, 31]. To what extend this statistical significance translates into clinical significance is not known. In the study by Basaki et al. [21], the IOS deviation was reported to be 116 (± 94) μm as compared to 56 (± 29) μm for the conventional impression procedure but the calculation was performed on the polyurethane milled casts that were produced from the digital impressions. Therefore, additional deviations in the milling process may have aggravated this discrepancy. In a recent in vivo study by Alsharbaty et al. [32] the authors reported statistically significant differences in accuracy between the conventional and the digital impression of partially edentulous sites with 2 adjacent implants, although clinical significance could not be concluded according to the authors.

### IOS accuracy compared to conventional impression accuracy in completely edentulous arches with multiple implants

Complete-arch IOS accuracy of multiple implant impressions has been studied intensively in the past 5 years. The conventional method, utilizing elastomeric impression materials and multiple implant impression post splinting, has been the gold standard against which the accuracy of various scanners was tested (Table 4). Current evidence on the superiority of one technique over the other is inconclusive. There is available research postulating that IOS of complete edentulous arches with 5 or 6 implants is either equally or statistically significantly more accurate than conventional elastomeric impressions taken using impression post splinting and an open custom tray approach [33–35].This finding is independent of the scanner used, as different IOS technologies such as confocal microscopy and active triangulation have been tested in the aforementioned studies. Impression material type is also non-contributory, as both polyether (PE) and polyvinylsiloxane (PVS) high accuracy elastomeric materials were used. In contrast, there is also available evidence supporting the significant statistical superior accuracy of the splinted, open-tray, conventional elastomeric impression technique over the IOS impression for complete-arch implant rehabilitation [36–38]. Again, this finding was irrespective of IOS device and impression material used. This lack of consensus can be attributed to factors such as the study design, the different IOS device software and hardware used or the statistical analysis employed but it is unclear whether statistical significance translates into clinical significance.

There appears to be scientific evidence in the available literature, however, regarding the superiority of the digital intraoral scanning method in relation to the conventional, non-splinted elastomeric impression technique using either an open or a closed custom tray for complete-arch impressions [39, 40]. In a study by Rech-Ortega et al. [40], the authors stated that despite the higher accuracy of the digital scanning method, both techniques exhibited a deterioration when more than 4 implants were involved in the scanning scope. Alikhasi et al. [39] reported that the digital impression technique was statistically more accurate than both the direct (open tray) and the indirect (closed tray) conventional elastomeric impression method.

Besides the conventional and the complete digital workflow for edentulous arches with multiple implants, there is always the option of producing a 3D-printed or milled cast from the IOS impression and using this as the master model. Research on the accuracy of such models produced using rapid prototyping techniques is scarce (Table 5). Papaspyridakos et al. [41] in an in vitro study on the accuracy of 4 implant analogue positions in SLA (Stereolithography) casts produced through IOS scans, concluded that the mean deviation of the printed casts was 59 (± 16) μm. The implant analogue 3d deviations were statistically significantly different from the master model, but still within a clinically acceptable range according to the authors [41]. In another in vitro study, Revilla-Leon et al. [42] tested several 3D-printing technologies for the production of a completely edentulous maxillary cast with 7 implants. The authors reported that not all production methods led to results comparable to the conventional gypsum master model in terms of accuracy. Digital light processing (DLP) and Polyjet 3D printing technologies with specific 3D printers showed comparable accuracy to the stone model. Implant analogue deviations ranged from 21 (± 16) μm (Polyjet) to 27 (± 20) μm (DLP).

### Factors influencing IOS accuracy in fixed implant-supported restorations

Several in vitro studies have been conducted comparing different scanner devices regarding both partial [43–47] and complete-arch [23, 44, 48–54] accuracy. Digital scans from the various IOS devices were compared for trueness and precision against the scans from a highly accurate reference laboratory scanner. Evidence suggests that scanner type and generation can influence scanning accuracy as some scanner devices exhibited higher precision (low standard deviation) and higher complete-arch scanning accuracy compared to others. Nevertheless, the majority of newer generation scanners produce complete-arch accuracy values less than the maximum 150 μm threshold, currently accepted in clinical practice [45, 55–58].

Several clinical factors contributing to the global deviations in complete-arch intraoral scanning have been identified and studied in the literature. Operator experience is one clinical parameter that has been reported to influence scanning accuracy in a study utilizing an Active Wavefront Sampling (AWS) technology scanner (Lava COS) [59] but more recent studies with newer generation scanners using both AWS and Confocal Microscopy technology failed to verify this finding, or identify the clinically relevant level of operator experience [48, 60, 61].

Implant angulation is another clinical factor that has been extensively studied for its effect on both partial and complete-arch implant digital impression accuracy. In the vast majority of studies, where single-part all-PEEK scanbodies were used as scanning posts for both partial [21, 24, 25] and complete-arch [33, 39, 48, 59, 62, 63] digital impressions, scanbody angulation did not affect scan accuracy. In the contrary, in a study by Arcuri et al. [61], the authors reported that complete-arch scan accuracy was, indeed, influenced by scanbody angulation. This finding may be attributed to the material of the scanbodies themselves. The PEEK-titanium scanbodies that were used in the study, presented the worst overall accuracy results compared to the all-PEEK and the titanium scanbodies they were compared against, possibly due to the interlocking between the two parts. In another in vitro study by Lin et al. [64], accuracy of the 2 implant analogues position in partial, milled polyurethane casts fabricated digitally following IOS, was found to be influenced by minor implant angulation (0–15 degrees) but not by major implant angulation (30–45 degrees). The authors reported that it is unclear whether the design of the two-piece scanbodies used in the study attributed to this result.

Further-on, regarding the design characteristics of scanbodies, their influence in scan accuracy has been tested in both partial and complete-arch digital implant impressions. The refractory and reflective indexes of all-PEEK scanbodies have been reported to be beneficial for complete-arch scan accuracy [44, 61]. Additionally, implant placement depth has not been reported to play a detrimental role in complete-arch IOS accuracy [48, 59] assuming the visible part of the scanbody can provide adequate reference points for IOS registration [62]. Therefore, using scanbodies of adequate length for optimum scan accuracy is indicated [57]. Cylindrical scanbodies with smoother surfaces have also been reported to facilitate IOS digitization by producing less noise as opposed to scanbodies with irregular shape [65–67]. Recent research has also highlighted the importance of certain features related to scanbody manufacturing tolerances and their effect on the accuracy of the digital IOS impression. Schmidt et al. [68] have reported significant differences in design characteristics such as length and diameter between implant scanbodies of the same manufacturer. These tolerances may affect the accurate transfer of implant position and therefore contribute to the final prosthetic misfit. In addition, Mangano et al. [69] have reported on the congruence between the IOS mesh file and the CAD library file of scanbodies when scanned with different IOS devices. Certain scanners seem to digitize the shape of the scanbody more closely to the actual CAD library file compared to others. Finally, implant scanbody reusability is another important parameter that needs to be consider. Limited evidence regarding all-PEEK scanbodies suggest that using them up to ten consecutive times does not impact on transfer accuracy [70].

Lighting conditions during a scanning session have recently been reported to influence global scanning accuracy. Research has shown that each IOS device scans more accurately in specific lighting conditions [71, 72] that correlate to its inherent image acquisition technology. Regardless of this technology however, precise superimposition or stitching of successive images is imperative for accurate scan results. This process is known to produce dimensional discrepancies that are directly related to both the scanning scope and the interimplant distance. Its effect is multiplied in complete-arch edentulous jaws with limited reference points and landmarks among multiple implants as well as in the mandibular posterior area where scanner tip access is compromised due to tongue movement and limited space [73, 74]. Studies on partial-arch digital scan accuracy have shown that when the range of scan and interimplant distance increased, the scanning accuracy decreased [43, 45, 46, 54]. Moreover, increasing scanning range and interimplant distance have also been reported to influence complete-arch scan trueness and precision [35, 40, 50, 60, 62, 75], although the minimum number of installed implants for an accurate digital impression has not yet been investigated. The main issue with multiple implant scanning in fully edentulous arches remains the difficulty in predictable scanning of the soft tissue between the fixtures themselves. Mizumoto et al. [75] in a recent in vitro study have reported that in the completely edentulous maxilla with 4 installed implants, including scanning data from the palate did not result in statistically significant higher accuracy. In an effort to minimize discrepancies when scanning edentulous sites among multiple implants, Iturrate et al. [76, 77] have investigated the in vitro effectiveness of using an auxiliary geometric device (AGD) firmly attached onto the implant scanbodies. The authors reported statistically significantly higher accuracy when the AGD was used regardless of the IOS scanner tested. Huang et al. [38] in an in vitro study have also reported improved complete-arch accuracy when modified, interconnected scanbodies on 4 implants were used. Motel et al. [65] in a recent in vitro study reported that scanning for a partial edentulous site with 3 adjacent implants in a single step (implant position scan only) led to more accurate results compared to scanning in two steps (emergence profile scan and implant position scan). The authors attributed this to the superimposition discrepancies that occur when the two scans are aligned in the scanner software. Finally, Alikhasi et al. [39] reported that the type of implant connection (internal or external) did not influence complete-arch scan accuracy using a confocal microscopy scanner in a maxillary edentulous jaw with 4 implants.

### Success and survival of monolithic single and multiple implant restorations manufactured using the direct digital workflow

Clinical studies regarding implementation of the complete digital workflow have been reported in the literature (Table 6) with the focus being mainly on the rehabilitation of single posterior implants following a digital intraoral impression procedure with or without the fabrication of a 3D-printed or milled master cast. Monolithic zirconia crowns and monolithic lithium disilicate crowns have been studied individually or compared to each other or to a metal-ceramic counterpart for success and survival.

In the available literature on monolithic zirconia crowns, success ranged from 92 to 100% and survival ranged from 97.5 to 100% for a follow-up of 1–3 years [78–81]. Technical complications such as ill-fitting crown on a prefabricated abutment, fracture of a cusp, infra-occlusion, inferior aesthetics and crown de-cementation were reported. Biological complications were minimal. A recent in vivo study by Lerner et al. [82] also reported very promising results for monolithic zirconia crowns fabricated on hybrid zirconia abutments following an IOS procedure. Success and survival rates after a mean of 3 years of follow-up were 91.3% and 99% respectively with a 1.9% biologic and 5.7% prosthetic incidence rate.

Available literature on monolithic lithium disilicate CAD-CAM crowns also shows encouraging results. Short-term in vivo studies exhibited a success rate of 89–100% and a survival rate of 100% for a follow-up of 2–3 years [5, 81]. Technical complications such as minor chippings were observed. Biological complications were again minimal.

Joda and coworkers reported on a cohort of patients rehabilitated with single-implant, porcelain-layered zirconia crowns following an IOS impression and a digital model fabrication process [83, 84]. At 3 years follow-up, both the success and survival rates were 100% and the patients reported high levels of satisfaction [84]. After 5 years of function however, one implant was lost leading to a success/survival rate of 95%. Mean bone loss around the implants increased significantly by 0.23 mm mesially and 0.17 mm distally compared to baseline [83].

A recent in vivo study reported on the fit of 2,3 and 4-units zirconia fixed partial dentures on 2 implants following a complete digital workflow [85] (Table 7.) The authors claimed that interimplant angulation exceeding 10 degrees could negatively influence the passive fit of the restorations as opposed to their counterparts, fabricated through a conventional workflow. Interimplant distance, on the other hand, exhibited no significant effect on passive fit of either group.

With regard to time efficiency of the digital workflow, several studies have reported statistically significant shorter clinical and laboratory working times for the complete digital, compared to a semi-digital or conventional workflow for single-implant rehabilitation [79, 86–88]. Regarding patient satisfaction, evidence also supported the significantly superior acceptance of the digital workflow in terms of comfort and ease of the IOS impression procedure compared to the conventional elastomeric impression [88, 89], although the final aesthetic outcome of monolithic zirconia restorations has been reported to be inferior to their porcelain-layered zirconia counterparts [78].

Within the scope of this review, no studies on the complete direct digital workflow for rehabilitation of multiple implants in edentulous arches were identified. Systematic reviews on the success and survival of implant-supported, zirconia complete fixed dentures fabricated through a conventional impression workflow, suggest that the use of monolithic or minimally veneered zirconia frameworks may help eliminate frequent complications encountered with veneering porcelain chipping [90, 91]. Minimal buccal veneering can also aid in solving the aesthetic problem often encountered with such designs but this restorative option has yet to be tested within the concept of the direct digital workflow.

## Conclusions

Based on this literature review, the following can be concluded:

The vast majority of identified studies were in vitro and this limited their clinical significance. Important clinical factors such as scanning accuracy and prosthesis’s misfit and their effect on technical or biological complications can only be studied effectively in longitudinal in vivo studies. For the single and short span implant sites, the IOS accuracy was high and the deviations in the position of the virtual implant fell within the acceptable clinical limits. When a semi-digital approach was elected, higher deviations in the position of the implant platform could be expected due to accumulated discrepancies in the 3D printing or milling fabrication process of the master model.

In the complete edentulous arch with multiple implants, there was no consensus regarding the superiority of the conventional, splinted, custom tray impression procedure compared to the IOS impression. On the contrary, digital complete-arch impressions were more accurate than conventional, non-splinted, open or close tray impressions. 3D-printing of the master model could induce further discrepancies in the digital workflow depending on the printing technology and materials used.

Newer generation scanners exhibited complete-arch deviation levels below the current acceptable threshold. Operator experience was not an influencing factor for complete-arch accuracy with newer scanners but critical experience level is yet to be determined. Lighting conditions during scanning can influence IOS device accuracy.

All-PEEK, one-part scanbodies with cylindrical shape, smooth surfaces and adequate length were preferred. Implant angulation did not influence IOS accuracy when scanbodies with the above features were used. Both scanbody manufacturing tolerances and congruence between scanbody IOS mesh and CAD file have been shown to influence scan trueness and precision.

Increasing scanning range and inter-implant distance can influence scan accuracy. Using auxiliary removable devices and interconnecting the scanbodies making sure not to disrupt their shape and size for correct digital registration, showed promising results. Limited evidence also suggested that implant connection type did not influence scan accuracy.

Regarding the complete digital workflow, for single implants cases, monolithic restorations exhibited high success and survival rates with minor technical complications for short to medium follow-up periods (3–5 years). Patient acceptance and total clinical and laboratory time efficiency has also been reported to be high. For multiple implants, this workflow has not yet been documented adequately for clinical use. Future studies on outcome measures such as patient acceptance, time efficiency, and technical and biological complications of multiple implant-supported prostheses should be conducted to draw clinical conclusions.

## Data Availability

Not applicable.

## Abbreviations

CNC: Computer numerically controlled
CEREC: Chairside Economical Restoration of the Esthetic Ceramics
IOS: Intraoral scanner
CAD: Computer assisted design
CAM: Computer assisted manufacturing
PEEK: Polyetheretherketone
PE: Polyether
PVS: Polyvinylsiloxane
SLA: Stereolithography
DLP: Digital light processing
AWS: Active wavefront sampling
AGD: Auxiliary geometric device
FPD: Fixed partial dentures
RP: Rapid prototyping
